# Socioeconomic deprivation and maternal mental health in developing countries: a multicenter study of postpartum depression and post-traumatic stress disorder

**DOI:** 10.3389/fgwh.2026.1803491

**Published:** 2026-07-30

**Authors:** Saad Zbiri, Carine Milcent

**Affiliations:** 1 Mohammed VI International School of Public Health, Mohammed VI University of Sciences and Health (UM6SS), Casablanca, Morocco; 2 Laboratory of Public Health, Health Economics, and Health Management, Mohammed VI University of Sciences and Health (UM6SS), Casablanca, Morocco; 3 Institute for Healthcare System Analysis (IA2S), Paris, France; 4 Paris-Jourdan Sciences Economiques, French National Center for Scientific Research (CNRS), Paris, France; 5 Paris School of Economics (PSE), Paris, France

**Keywords:** low- and middle-income countries, maternal mental health, postpartum depression, post-traumatic stress disorder, socioeconomic deprivation

## Abstract

Despite growing research on maternal mental health, evidence from low- and middle-income countries, particularly in Africa, remains limited and fragmented. We provide context-specific evidence in the post-COVID era and address an important gap in the literature on socioeconomic vulnerability and maternal mental health. This study investigates the effect of socioeconomic deprivation on postpartum depression and post-traumatic stress disorder among postpartum women using validated assessment tools. Maternal mental health outcomes are assessed using the Edinburgh Postnatal Depression Scale (EPDS) and the Peritraumatic Distress Inventory (PDI), while socioeconomic deprivation is measured using the multidimensional EPICES score. Our study is a multicenter cross-sectional observational study conducted in three hospitals in Casablanca and Rabat, Morocco. A total of 527 women who consent to participate are consecutively recruited at the end of their maternity stay between June 2024 and June 2025. Descriptive analyses show that 26.9% (95% CI: 23.3–30.9%) of women screen positive for postpartum depression and 34.2% (95% CI: 30.2–38.3%) for post-traumatic stress disorder, with 73.2% (95% CI: 69.3–76.9%) classified as socioeconomically deprived. Multivariate logit models reveal that socioeconomic deprivation, measured using both the continuous EPICES score and the dichotomized EPICES indicator, independently increases the risk of postpartum depression (*β* = 0.028, *p* < 0.01; *β* = 0.631, *p* < 0.05) and post-traumatic stress disorder (*β* = 0.030, *p* < 0.01; *β* = 1.018, *p* < 0.05). Sensitivity analyses using detailed socioeconomic characteristics confirm the robustness of these effects. The findings underscore the significant impact of socioeconomic disadvantage on maternal mental health and highlight the need for integrated interventions addressing both social and medical determinants of postpartum psychological well-being in developing-country settings.

## Introduction

Maternal mental health is a critical population health concern, with perinatal mental health conditions affecting up to 20% of pregnant or postpartum women and recognized as leading contributors to preventable maternal deaths during pregnancy and the first postpartum year (1). This issue is particularly acute in developing countries, where healthcare resources are limited and socioeconomic stressors are pervasive. Global and regional analyses show that, although the leading causes of maternal death are hemorrhage, indirect obstetric causes, and hypertensive disorders, maternal mental health conditions also contribute substantially to preventable maternal mortality and are often underreported (2). Most deaths occur postpartum, especially in low- and lower-middle-income countries (LMICs), underscoring both the heavy burden of maternal mortality and the urgent need to address the broader clinical and nonclinical risk factors shaping adverse maternal outcomes (1, 2).

Globally, postpartum depression affects approximately 10%–20% of women, with higher prevalence reported in LMICs and African settings (3). In LMICs, perinatal depression affects approximately one in four women, with a pooled prevalence of 24.7% (4). In Africa, reported prevalence estimates range from 6.9% to 43%, highlighting the substantial burden and heterogeneity of postpartum depression across the continent (5). Comparable findings have been reported in Morocco, where postpartum depression prevalence remains substantial among postpartum women (6, 7). In addition to postpartum depression, childbirth-related post-traumatic stress disorder may develop following actual or perceived traumatic childbirth experiences and is also increasingly recognized as a major maternal mental health concern, affecting an estimated 3%–6% of women in community samples and up to 15%–20% in high-risk populations (8). Studies from LMICs suggest that prevalence rates may be similar or even higher, ranging from 3% to 20% (9). Similarly, a high prevalence of postpartum post-traumatic stress disorder has been documented in Morocco (10). Together, these findings highlight the substantial burden of postpartum depression and post-traumatic stress disorder across LMIC and African settings, while underscoring the limited and heterogeneous nature of the available evidence (11).

Perinatal mental disorders generate substantial adverse consequences for mothers, infants, and families. Maternal depression has been shown to negatively affect maternal health, family well-being, and children's emotional and social development, while contributing to the intergenerational transmission of poverty (12). Maternal depression is further associated with adverse long-term socioeconomic outcomes, including lower household income, increased unemployment, and greater material hardship (13). In Africa, perinatal depression has been linked to adverse birth and infant health outcomes, including preterm birth, low birth weight, malnutrition, and febrile illnesses (14). Childbirth-related post-traumatic stress disorder has likewise been associated with adverse maternal outcomes, including persistent psychological distress, impaired maternal well-being, and difficulties in mother-infant bonding. It may also negatively affect breastfeeding practices, infant development, and family functioning (8, 9). Overall, the evidence underlines the wide-ranging impact of postpartum depression and post-traumatic stress disorder on maternal, infant, and family well-being and supports the urgent need for focused research and evidence-based interventions.

Socioeconomic determinants, such as income, education, access to healthcare, and social support, are key health factors that can directly impact mental health (15). Scientific literature shows that individuals facing socioeconomic disadvantage experience higher rates of mental health disorders. Previous studies have shown that socioeconomic deprivation is associated with poorer mental health outcomes and contributes to maternal morbidity and mortality through multiple social, economic, and health-system pathways (16–18). Yet, data from developing countries remain limited and fragmented (19). Studies from LMICs suggest that neighborhood deprivation, poverty, and other forms of socioeconomic disadvantage are associated with worse mental health outcomes, although methodological limitations and substantial heterogeneity persist (19, 20). Socioeconomic deprivation may contribute to postpartum depression and post-traumatic stress disorder through multiple pathways, including financial stress, reduced access to healthcare, inadequate social support, poorer living conditions, and increased exposure to adverse life events. These factors may heighten psychological vulnerability during pregnancy and the postpartum period and impair maternal coping capacities (11, 18, 20). In Africa, maternal mental health disorders are strongly shaped by socioeconomic and poverty-related factors operating at the individual, family, social, and environmental levels, yet high-quality evidence remains scarce (11). Similarly, evidence from Morocco remains limited and is largely based on relatively small studies, although it suggests an important role of socioeconomic vulnerability factors in postpartum depression and post-traumatic stress disorder (6, 10). These results support further rigorous, context-specific, and high-quality research in LMICs to better understand how socioeconomic deprivation contributes to maternal mental health outcomes.

Examining maternal mental health across developing-country settings is crucial for identifying at-risk populations, guiding timely interventions, and informing context-specific public health strategies, a need that has intensified with the COVID-19 pandemic. Evidence indicates that psychological distress among perinatal women increased substantially during the COVID-19 pandemic, with higher levels of depression and post-traumatic stress symptoms reported across settings (21). In addition, pandemic-related disruptions in healthcare delivery and childbirth experiences, as well as increased social and economic pressures on mothers, may have further contributed to adverse maternal mental health outcomes (22). Studies conducted during the pandemic also report a particularly high burden of perinatal mental health disorders in Africa and highlight the need for further research on contributing factors and clinical outcomes (23). The post-COVID period provides an important context for examining maternal mental health and its determinants.

Despite increasing recognition of maternal mental health as a major public health issue, important gaps remain in the literature. First, evidence from LMICs, particularly in African settings, remains limited. Second, most studies have focused on either postpartum depression or post-traumatic stress disorder separately, with few examining both outcomes simultaneously. Third, little is known about the role of multidimensional socioeconomic deprivation in shaping maternal mental health outcomes, particularly when assessed using validated measures. Finally, multicenter studies conducted in African postpartum populations remain scarce. To address these gaps, this multicenter study, conducted in Morocco in the post-COVID context, examines maternal mental health, including postpartum depression and post-traumatic stress disorder, and evaluates how socioeconomic deprivation, assessed using the validated multidimensional EPICES score, may drive their occurrence.

## Methods

This study is a multicenter analytical cross-sectional study conducted in three hospitals (two public and one private) in Casablanca and Rabat, Morocco, between June 2024 and June 2025. Data sources combine medical record reviews with direct patient interviews. Medical records are accessed by the research team within the participating hospitals following institutional authorization and ethical approval procedures, and face-to-face patient interviews are conducted by trained research staff. Medical records are used to ensure the accuracy and completeness of clinical and obstetrical information and to minimize recall and information bias, while face-to-face interviews are used to collect additional information not available in medical records. We collect maternal demographic characteristics, medical history (including psychiatric history), pregnancy characteristics, delivery characteristics, postpartum characteristics, and socioeconomic characteristics of the mother and her partner. In parallel, standardized instruments assess maternal mental health and socioeconomic vulnerability. Questionnaires are administered through face-to-face interviews conducted by trained members of the research team to ensure comprehension and minimize missing data. All assessments are conducted at the end of the maternity stay during the immediate postpartum period, before hospital discharge. Postpartum depression is measured with the Edinburgh Postnatal Depression Scale (EPDS), a validated 10-item self-report questionnaire widely used for screening (24). Scores range from 0 to 30, with higher scores reflecting more severe depressive symptoms. In accordance with previous validation studies and international recommendations, a cutoff score of ≥13 identifies women at risk (25). Post-traumatic stress disorder is assessed with the Peritraumatic Distress Inventory (PDI), a 13-item self-report scale that captures distress experienced during or immediately after a traumatic event, including for childbirth-related trauma (26). Scores are continuous, with higher values indicating greater distress; based on previous validation studies, a cutoff score of ≥15 identifies women at risk. Socioeconomic vulnerability is evaluated with the EPICES score (*Évaluation de la Précarité et des Inégalités de santé dans les Centres d'Examens de Santé*), an individual multidimensional indicator of deprivation ranging from 0 (no deprivation) to 100 (maximum deprivation) (27). This reliable index has been validated in a population of women who have just given birth (28). Evidence indicates that this indicator can reliably assess socioeconomic deprivation in various settings (29). A threshold of ≥30 is commonly used to define socioeconomic deprivation (27). While several studies propose alternative cutoffs, we adopt the most widely applied threshold and also retain the variable in its continuous form. All data are collected during the immediate postpartum period.

A total of 527 women who recently gave birth are recruited at the end of their maternity stay, with 460 having complete information on socioeconomic status. Participants are consecutively recruited among all postpartum women aged 18 years and older admitted to the participating hospitals during the study period who agreed to participate. Several categories of variables are collected from medical records and patient interviews:

- Demographic variables: maternal age, parity (number of previous deliveries), and number of children in the household;
- Socioeconomic status variables: maternal familial situation (categorized as single, including divorced, separated, and widowed), maternal health coverage (classified as uninsured if no public or private insurance, or other types of coverage), maternal education level (high: more than 2 years of university education; intermediate: completed high school up to 2 years of university education; low: primary, middle, or high school), maternal employment status (considered unemployed if not engaged in any salaried full-time, part-time, or self-employed work), and partner employment status (considered unemployed under the same criteria);
- Socioeconomic deprivation variables: EPICES score and positive screening;
- Medical history variables: previous obstetric disorders (premature or low-weight birth, fetal or neonatal death, cesarean delivery), previous mental disorders (all psychological disabilities including depression), and substance use (tobacco or alcohol);
- Pregnancy variables: multiple pregnancy (two or more fetuses), abnormal fetal presentation (breech or transverse), and pregnancy-related diseases (including arterial hypertension, diabetes, infection, intrauterine growth restriction, anemia, etc.);
- Delivery variables: term at delivery (gestational age: preterm < 37 weeks, full-term = 37 to 41 weeks, post-term ≥41 weeks), mode of delivery (normal delivery, instrumental delivery with forceps or vacuum, and cesarean delivery), episiotomy use, analgesia use, delivery complications (including dystocia, obstructed labor, hemorrhage, perineal tear, etc.), and private sector delivery;
- Early postpartum variables: newborn weight (low < 2,500 grams, normal = 2,500 to 4,000 grams, high >4,000 grams), newborn vitality (Apgar score at 5 min: low < 4, intermediate = 4 to 7, normal >7), artificial feeding, maternal postpartum diseases (including infection, hemorrhage, thromboembolic event, breast complication, etc.), neonatal diseases (including respiratory distress, jaundice, infection, congenital anomaly, etc.);
- Maternal mental health outcomes: postpartum depression (EPDS score and positive screening), and post-traumatic stress disorder (PDI score and positive screening).

The analysis includes both descriptive and analytical components. First, descriptive statistics are computed for all variables. Continuous variables are presented as means with standard deviations, and categorical variables as counts and percentages. To assess the effect of socioeconomic deprivation on maternal mental health, we estimate logit regression models (30). The dependent variables are maternal mental health outcomes, specifically postpartum depression (dichotomized at the threshold of 13 on the EPDS) and post-traumatic stress disorder (dichotomized at the threshold of 15 on the PDI). The main exposures of interest are the EPICES score, considered both as a continuous variable and dichotomized at the threshold of 30. We control for demographic variables (maternal age, parity, number of children in the household); medical history variables (previous obstetric or mental disorders, substance use); pregnancy variables (multiple pregnancy, abnormal fetal presentation, pregnancy-related diseases); delivery variables (term at delivery, mode of delivery, episiotomy, analgesia, delivery complications, private sector delivery); and early postpartum variables (newborn weight and vitality, artificial feeding, maternal and neonatal diseases). We perform both univariate and multivariate analyses. Multivariate models include variables with *p* < 0.10 in univariate analyses to avoid overfitting and limit model complexity. We also verified our results by estimating models that included the full set of observable variables. Coefficient estimates with standard errors are reported. Sensitivity analyses are conducted using detailed socioeconomic status variables, including maternal familial situation, health insurance coverage, education level, and both parental employment status. Statistical significance is reported at three thresholds: 1% (*p* < 0.01), 5% (*p* < 0.05), and 10% (*p* < 0.10) (31). All analyses are conducted using Stata statistical software (32).

The study is conducted with the approval of the administrative authorities of the participating hospitals. Informed consent is obtained from all participants prior to enrollment, and only women providing explicit consent are included. The research protocol is reviewed and approved by the Ethics Committee of Mohammed VI University of Sciences and Health (reference: CE/UM6SS/04/23). All procedures comply with ethical standards for research involving human participants.

## Results

A total of 527 women who have recently given birth are included in the study sample.

### Description of the study population

As shown in Table 1, the mean age of participants is 29.4 years [standard deviation (SD) = 6.3; range: 18–46], with a mean parity of 1.4 (SD = 1.3; range: 0–13) and an average of 1 child living in the household (SD = 1.1; range: 0–7). Among the 460 women with complete socioeconomic information, 1.5% are single mothers, 26.5% are uninsured, 11.7% have a high level of education, 21.1% an intermediate level, and 67.2% a low level. In addition, 82.6% of mothers are not employed, compared with 6.5% of partners. Socioeconomic vulnerability is high among participants, with a mean EPICES score of 47.6 (SD = 23.1; range: 0–100); 73.2% (95% CI: 69.3–76.9%) of women are classified as socioeconomically deprived.

**Table 1:** Descriptive statistics.

Variables	*N* (observations)	*n* (%) or mean ± SD
Demographics		
Age	527	29.37 ± 6.29
Parity	527	1.42 ± 1.27
Children in the household	527	1.04 ± 1.09
Socioeconomic status		
Single mother	460	7 (1.52)
Uninsured mother	460	122 (26.52)
Maternal education	460	
High		54 (11.74)
Intermediate		97 (21.09)
Low		309 (67.17)
Non-working mother	460	380 (82.61)
Non-working partner	460	30 (6.52)
Socioeconomic deprivation		
EPICES score	527	47.61 ± 23.11
Positive EPICES score	527	386 (73.24)
Medical history		
Previous obstetric disorder	527	215 (40.80)
Previous mental disorder	527	96 (18.22)
Substance use	527	6 (1.14)
Pregnancy		
Multiple pregnancy	527	14 (2.66)
Abnormal fetal presentation	527	40 (7.59)
Pregnancy disease	527	219 (41.56)
Delivery		
Term at delivery	527	
Preterm		52 (9.87)
Full-term		402 (76.28)
Post-term		73 (13.85)
Mode of delivery	527	
Normal vaginal delivery		114 (21.63)
Instrumental vaginal delivery		223 (42.32)
Cesarean delivery		190 (36.05)
Episiotomy	527	196 (37.19)
Analgesia	527	386 (73.24)
Delivery complication	527	78 (14.80)
Private sector delivery	527	110 (20.87)
Early postpartum		
Newborn weight	527	
Low		42 (7.97)
Normal		461 (87.48)
High		24 (4.55)
Newborn vitality	527	
Normal		506 (96.01)
Intermediate		7 (1.33)
Low		14 (2.66)
Artificial feeding	527	34 (6.45)
Maternal disease	527	20 (3.80)
Neonatal disease	527	139 (26.38)
Postpartum depression		
EPDS score	527	9.03 ± 6.03
Positive EPDS score	527	142 (26.94)
Post-traumatic stress disorder		
PDI score	527	13.16 ± 11.32
Positive PDI score	527	180 (34.16)

Means with standard deviations are reported for continuous variables, and counts with percentages for categorical variables.

EPDS, edinburgh postnatal depression scale; PDI, peritraumatic distress inventory; EPICES, socioeconomic deprivation index (*Évaluation de la Précarité et des Inégalités de santé dans les Centres d’Examens de Santé*).

Regarding medical history, 40.8% have a previous obstetric disorder, 18.2% report a prior mental disorder, and 1.1% have a history of substance use. Pregnancy characteristics show that 2.7% of women have a multiple pregnancy, 7.6% an abnormal fetal presentation, and 41.6% a pregnancy-related disease. Concerning delivery outcomes, 9.9% of births are preterm, 76.3% full-term, and 13.9% post-term. The mode of delivery is normal vaginal in 21.6% of cases, instrumental vaginal in 42.3%, and cesarean in 36.1%. Episiotomy is performed in 37.2% of deliveries, analgesia is administered to 73.2%, and 14.8% experience delivery complications. A fifth of deliveries (20.9%) take place in private sector facilities. Early postpartum characteristics indicate that 7.9% of newborns have low birth weight, 87.5% normal weight, and 4.6% high birth weight. Newborn vitality is normal in 96% of cases, intermediate in 1.3%, and low in 2.7%. Artificial feeding is reported in 6.5%. Maternal disease in the early postpartum period occurs in 3.8%, while 26.4% of newborns experience neonatal disease.

Regarding maternal mental health, the mean EPDS score is 9.0 (SD = 6.0; range: 0–28), with 26.9% (95% CI: 23.3–30.9%) of women screening positive for postpartum depression. The mean PDI score is 13.2 (SD = 11.3; range: 0–52), and 34.2% (95% CI: 30.2–38.3%) of women screen positive for post-traumatic stress disorder.

### Socioeconomic deprivation and postpartum depression

We first examine the effect of socioeconomic deprivation on postpartum depression using univariate and multivariate logit regression models in Table 2. In univariate analyses, several factors significantly increase the probability of postpartum depression, including previous mental disorder, substance use, abnormal fetal presentation, preterm delivery, instrumental vaginal delivery, analgesia, low newborn weight, low or intermediate newborn vitality, artificial feeding, maternal postpartum disease, neonatal disease, and higher EPICES scores.

**Table 2:** Effect of socioeconomic deprivation on postpartum depression.

Variables	Univariate analysis	Multivariate analysis: EPICES score	Multivariate analysis: Positive EPICES score
Demographics			
Age	0.041 (0.016)**	0.026 (0.022)	0.023 (0.022)
Parity	0.244 (0.078)***	0.011 (0.186)	0.012 (0.182)
Children in the household	0.310 (0.088)***	0.239 (0.217)	0.273 (0.212)
Socioeconomic deprivation			
EPICES score	0.027 (0.005)***	0.028 (0.006)***	
Positive EPICES score	0.702 (0.247)***		0.631 (0.317)**
Medical history			
Previous obstetric disorder	0.515 (0.198)***	0.419 (0.262)	0.328 (0.257)
Previous mental disorder	1.783 (0.240)***	1.850 (0.284)***	1.941 (0.279)***
Substance use	1.714 (0.872)**	2.743 (1.168)**	2.968 (1.122)***
Pregnancy			
Multiple pregnancy	0.083 (0.600)		
Abnormal fetal presentation	0.761 (0.336)**	0.228 (0.427)	0.372 (0.411)
Pregnancy disease	0.158 (0.198)		
Delivery			
Term at delivery			
Preterm	0.637 (0.304)**	0.334 (0.459)	0.221 (0.456)
Full-term	reference	reference	reference
Post-term	−0.325 (0.311)	−0.556 (0.380)	−0.464 (0.370)
Mode of delivery			
Normal vaginal delivery	reference	reference	reference
Instrumental vaginal delivery	−0.512 (0.252)**	0.419 (0.432)	0.393 (0.422)
Cesarean delivery	−0.376 (0.257)	0.620 (0.505)	0.349 (0.492)
Episiotomy	−0.201 (0.206)		
Analgesia	−0.472 (0.214)**	−0.241 (0.404)	−0.278 (0.396)
Delivery complication	0.361 (0.263)		
Private sector delivery	−0.282 (0.252)		
Early postpartum			
Newborn weight			
Low	1.113 (0.327)***	0.837 (0.485)*	0.822 (0.477)*
Normal	reference	reference	reference
High	0.226 (0.462)	0.722 (0.574)	0.511 (0.542)
Newborn vitality			
Normal	reference	reference	reference
Intermediate	−0.740 (1.085)	−2.177 (1.202)*	−2.662 (1.247)**
Low	1.968 (0.600)***	1.065 (0.766)	1.121 (0.760)
Artificial feeding	0.951 (0.359)***	1.458 (0.436)***	1.353 (0.433)***
Maternal disease	1.694 (0.480)***	1.265 (0.552)**	1.360 (0.543)**
Neonatal disease	0.501 (0.214)**	0.031 (0.273)	0.182 (0.270)
*N* (observations)	527	527	527

EPICES, socioeconomic deprivation index (*Évaluation de la Précarité et des Inégalités de santé dans les Centres d’Examens de Santé*).

Coefficient estimates from logit models are presented with standard errors in parentheses.

Postpartum depression is assessed using the EPDS (Edinburgh Postnatal Depression Scale).

* *P*-value <0.10.

** *P*-value <0.05.

*** *P*-value <0.01.

In multivariate analyses controlling for demographic, medical history, pregnancy, delivery, and early postpartum variables, previous mental disorder and substance use remain strong predictors of postpartum depression. Among early postpartum variables, low newborn weight, intermediate or low newborn vitality, artificial feeding, and maternal postpartum disease significantly affect the probability of postpartum depression.

Crucially, socioeconomic deprivation significantly impacts postpartum depression. Both the continuous EPICES score (*β* = 0.028, *p* < 0.01) and the dichotomized positive EPICES status (*β* = 0.631, *p* < 0.05) remain significant in multivariate models, indicating that higher deprivation increases the probability of postpartum depressive symptoms even after controlling for multiple confounders. Controlling for the full set of observable factors does not alter this result (see Supplementary Table A1 in the Supplementary Appendix). Overall, these findings highlight the multifactorial determinants of postpartum depression in this population, with socioeconomic vulnerability emerging as a key independent risk factor.

### Socioeconomic deprivation and post-traumatic stress disorder

We next assess the effect of socioeconomic deprivation on postpartum post-traumatic stress disorder using univariate and multivariate logit regression models in Table 3. In univariate analyses, older maternal age, previous obstetric or mental disorder, substance use, pregnancy-related disease, preterm delivery, instrumental or cesarean delivery, episiotomy, delivery in a private facility, high or low newborn weight, low newborn vitality, and maternal postpartum disease significantly increase the probability of post-traumatic stress symptoms.

**Table 3:** Effect of socioeconomic deprivation on post-traumatic stress disorder.

Variables	Univariate analysis	Multivariate analysis: EPICES score	Multivariate analysis: Positive EPICES score
Demographics			
Age	0.040 (0.015)***	0.047 (0.020)**	0.047 (0.020)**
Parity	−0.096 (0.076)		
Children in the household	0.121 (0.084)		
Socioeconomic deprivation			
EPICES score	−0.013 (0.004)***	0.030 (0.008)***	
Positive EPICES score	−1.154 (0.204)***		1.018 (0.492)**
Medical history			
Previous obstetric disorder	0.679 (0.187)***	−0.188 (0.272)	−0.164 (0.268)
Previous mental disorder	1.463 (0.237)***	1.858 (0.310)***	1.987 (0.308)***
Substance use	2.291 (1.099)**	3.556 (1.419)**	3.801 (1.350)***
Pregnancy			
Multiple pregnancy	0.379 (0.548)		
Abnormal fetal presentation	0.385 (0.334)		
Pregnancy disease	0.873 (0.188)***	0.730 (0.258)***	0.577 (0.249)**
Delivery			
Term at delivery			
Preterm	0.726 (0.297)**	−0.138 (0.501)	−0.121 (0.489)
Full-term	reference	reference	reference
Post-term	−0.704 (0.308)**	−0.417 (0.395)	−0.392 (0.384)
Mode of delivery			
Normal vaginal delivery	reference	reference	reference
Instrumental vaginal delivery	−0.749 (0.259)***	−0.365 (0.552)	−0.310 (0.544)
Cesarean delivery	0.696 (0.245)***	0.105 (0.377)	0.003 (0.365)
Episiotomy	−1.184 (0.214)***	0.336 (0.537)	0.185 (0.532)
Analgesia	0.050 (0.208)		
Delivery complication	−0.324 (0.270)		
Private sector delivery	2.511 (0.259)***	3.869 (0.484)***	3.592 (0.551)***
Early postpartum			
Newborn weight			
Low	0.970 (0.326)***	0.411 (0.564)	0.460 (0.547)
Normal	reference	reference	reference
High	0.779 (0.420)*	1.115 (0.611)*	0.950 (0.575)*
Newborn vitality			
Normal	reference	reference	reference
Intermediate	0.447 (0.770)	−0.428 (1.190)	−0.700 (1.182)
Low	3.300 (1.042)***	2.378 (1.136)**	2.584 (1.153)**
Artificial feeding	−0.555 (0.415)		
Maternal disease	1.567 (0.497)***	1.948 (0.586)***	2.097 (0.584)***
Neonatal disease	0.194 (0.206)		
*N* (observations)	527	527	527

EPICES, socioeconomic deprivation index (*Évaluation de la Précarité et des Inégalités de santé dans les Centres d’Examens de Santé*).

Coefficient estimates from logit models are presented with standard errors in parentheses.

Post-traumatic stress disorder is assessed using the PDI (Peritraumatic Distress Inventory).

* *P*-value <0.10.

** *P*-value <0.05.

*** *P*-value <0.01.

In multivariate analyses controlling for demographic, medical history, pregnancy, delivery, and early postpartum variables, several factors remain independent predictors of post-traumatic stress disorder. Previous mental disorder, substance use, pregnancy-related disease, delivery in a private sector facility, low newborn vitality, and maternal postpartum disease increase the probability of post-traumatic stress symptoms.

Socioeconomic deprivation also significantly affects post-traumatic stress disorder. Both the continuous EPICES score (*β* = 0.030, *p* < 0.01) and the dichotomized positive EPICES status (*β* = 1.018, *p* < 0.05) remain significant, indicating that higher deprivation increases the probability of post-traumatic stress symptoms even after controlling for multiple confounders. The inclusion of all observable variables in the regression models does not affect this finding (see Supplementary Table A2 in the Supplementary Appendix). Overall, these results indicate that, similar to postpartum depression, socioeconomic vulnerability emerges as a key determinant of postpartum post-traumatic stress disorder symptoms, alongside medical and perinatal risk factors.

### Sensitivity analysis: socioeconomic status, postpartum depression and post-traumatic stress disorder

Finally, to assess the robustness of our findings, we perform sensitivity analyses using multivariate logit models including detailed socioeconomic status indicators, presented in Table 4. For postpartum depression, previous mental disorder, low newborn weight, and artificial feeding remain main predictors. Interestingly, being a single mother (*β* = 2.815, *p* < 0.05), low maternal education (*β* = 1.038, *p* < 0.10), and having a non-working partner (*β* = 1.136, *p* < 0.05) also independently impact the probability of postpartum depression. Other factors show weaker or non-significant effects. For post-traumatic stress disorder, previous mental disorder, delivery in a private sector facility, and low newborn vitality remain key predictors. Notably, having an uninsured mother (*β* = 0.702, *p* < 0.05), and a non-working partner (*β* = 1.513, *p* < 0.01) significantly affect the probability of post-traumatic stress disorder. Other variables do not significantly affect post-traumatic stress symptoms.

**Table 4:** Effect of socioeconomic status on postpartum depression and post-traumatic stress disorder.

Variables	Multivariate analysis: Postpartum depression	Multivariate analysis: Post-traumatic stress disorder
Demographics		
Age	0.040 (0.025)	0.078 (0.024)***
Parity	0.138 (0.212)	
Children in the household	0.087 (0.243)	
Socioeconomic status		
Single mother	2.815 (1.203)**	−0.230 (1.342)
Uninsured mother	0.366 (0.320)	0.702 (0.347)**
Maternal education		
High	reference	reference
Intermediate	0.539 (0.588)	−0.801 (0.582)
Low	1.038 (0.556)*	0.320 (0.612)
Non-working mother	0.172 (0.432)	0.555 (0.461)
Non-working partner	1.136 (0.517)**	1.513 (0.521)***
Medical history		
Previous obstetric disorder	0.264 (0.298)	−0.241 (0.308)
Previous mental disorder	2.233 (0.326)***	2.263 (0.358)***
Substance use	1.094 (2.219)	3.216 (2.249)
Pregnancy		
Abnormal fetal presentation	0.141 (0.497)	
Pregnancy disease		0.950 (0.296)***
Delivery		
Term at delivery		
Preterm	0.122 (0.533)	−0.571 (0.547)
Full-term	reference	reference
Post-term	−0.999 (0.513)*	−0.499 (0.482)
Mode of delivery		
Normal vaginal delivery	reference	reference
Instrumental vaginal delivery	0.621 (0.488)	−0.656 (0.618)
Cesarean delivery	0.698 (0.567)	−0.081 (0.432)
Episiotomy		0.362 (0.597)
Analgesia	−0.488 (0.456)	
Private sector delivery		3.680 (0.540)***
Early postpartum		
Newborn weight		
Low	1.266 (0.558)**	1.034 (0.643)
Normal	reference	reference
High	0.847 (0.601)	1.268 (0.657)*
Newborn vitality		
Normal	reference	reference
Intermediate	−2.591 (1.383)*	−0.646 (1.258)
Low	1.544 (0.889)*	2.681 (1.299)**
Artificial feeding	1.707 (0.506)***	
Maternal disease	1.313 (0.678)	1.146 (0.707)
Neonatal disease	0.114 (0.308)	
*N* (observations)	460	460

EPICES, socioeconomic deprivation index (*Évaluation de la Précarité et des Inégalités de santé dans les Centres d’Examens de Santé*).

Coefficient estimates from logit models are presented with standard errors in parentheses.

Postpartum depression is assessed using the EPDS (Edinburgh Postnatal Depression Scale).

Post-traumatic stress disorder is assessed using the PDI (Peritraumatic Distress Inventory).

* *P*-value <0.10.

** *P*-value <0.05.

*** *P*-value <0.01.

Even after controlling for all observable factors, the findings remain unchanged (see Supplementary Table A3 in the Supplementary Appendix). These sensitivity analyses confirm that maternal mental health outcomes are influenced not only by demographic, medical, and perinatal factors but also by socioeconomic conditions, reinforcing the robustness of the observed effects of socioeconomic deprivation on both postpartum depression and post-traumatic stress disorder.

## Discussion

This study quantifies the effect of socioeconomic deprivation on maternal mental health among women giving birth in Casablanca and Rabat, Morocco. Our findings demonstrate that socioeconomic vulnerability exerts a significant independent impact on both postpartum depression and post-traumatic stress disorder, even after controlling for demographic, medical history, pregnancy, delivery, and early postpartum-related determinants. Beyond clinical and obstetric factors, the social and economic context of mothers appears to play a crucial role in shaping postpartum psychological outcomes in developing-country settings.

The prevalence of postpartum depression (26.9%) and post-traumatic stress disorder symptoms (34.2%) in our sample aligns with rates reported in other LMICs. For postpartum depression, a recent systematic review reports a mean prevalence of 27.6%, with higher rates observed among vulnerable populations (33). Similarly, a meta-analysis from LMICs estimates a pooled prevalence of perinatal depression of 24.7%, with the highest rates reported in lower-middle-income countries (4). Another study indicates a global postpartum depression prevalence of 17.2%, with substantially higher rates observed in Southern Africa (34). For postpartum post-traumatic stress disorder, a study conducted in Ethiopia reports a prevalence of 21.6%, suggesting that trauma-related symptoms after delivery are relatively common in African settings (35). Our estimates are also comparable to previous findings from Morocco, where postpartum depression prevalence is reported at 18.7% in a clinical sample and 30% among women living in socially vulnerable conditions, while childbirth-related post-traumatic stress disorder prevalence reaches 28.6% among postpartum women (6, 7, 10). Several factors may account for the variability in prevalence estimates across studies, including differences in the timing of postpartum assessment, the screening instruments and the thresholds used (particularly EPDS and PDI cut-offs), study settings (community vs. hospital-based recruitment), and population characteristics such as socioeconomic vulnerability and obstetric risk profiles.

In our sample, 73.2% of women are classified as socioeconomically deprived, reflecting substantial social vulnerability among postpartum women in these settings. This prevalence is higher than what is typically reported in studies from high-income countries (28). However, these levels are consistent with patterns reported in more socioeconomically disadvantaged settings. A study conducted in socioeconomically contrasted French regions using the EPICES questionnaire reports that 53.2% of participants were classified as socially deprived, with higher EPICES scores observed in the French West Indies and French Guiana than in mainland France, confirming the validity of the EPICES score across diverse socioeconomic contexts (29). The higher prevalence of socioeconomic deprivation observed in our study may reflect differences in socioeconomic conditions, social protection systems, employment opportunities, and access to healthcare. In addition, our study focused on postpartum women attending public and private maternity facilities, a population that may experience specific socioeconomic vulnerabilities compared with the more heterogeneous populations included in previous studies.

Women with higher EPICES scores, reflecting multidimensional socioeconomic deprivation, show an increased probability of experiencing depressive and post-traumatic stress symptoms. As highlighted in the Introduction, few studies in LMICs examine maternal mental health in relation to socioeconomic disadvantage. In South Africa, socioeconomic disadvantage, including low socioeconomic status and childhood trauma, has been associated with an increased risk of maternal depression (36). Similarly, a study conducted in Iran finds that low income, limited education, household debt, inadequate insurance coverage, and poor housing conditions are associated with poorer postpartum mental health outcomes, including depression and stress (37). Differences in the magnitude and significance of socioeconomic determinants across studies may be related to variations in measurement tools, socioeconomic indicators, healthcare systems, and cultural contexts. These contextual differences may influence both the expression of maternal mental health symptoms and the pathways through which socioeconomic disadvantage affects psychological well-being.

In addition to socioeconomic factors, clinical and perinatal variables significantly predict maternal mental health. A previous mental disorder is the strongest predictor. Maternal disease, low newborn weight or vitality, and artificial feeding increase the risk of postpartum depression. Previous studies have identified a history of mental illness, stressful life events, lifetime abuse, childcare stress, chronic health conditions, preeclampsia, gestational diabetes, exposure to second-hand smoke, and sleep disturbances as important risk factors for perinatal depression (33). Other evidence further highlights the role of premenstrual syndrome, experiences of violence, unintended pregnancy, cesarean delivery, preterm birth, anemia, and vitamin D deficiency (38). Regarding childbirth-related post-traumatic stress disorder, delivery in private sector facilities, low newborn vitality, and maternal or neonatal disease are significant predictors. Previous studies identify prior trauma, mental health problems, negative birth experiences, obstetric complications, and inadequate support during childbirth as key determinants of postpartum post-traumatic stress disorder (9). Additional evidence highlights the contribution of obstetric interventions, obstetric violence, and antenatal depression or anxiety (39).

Sensitivity analyses examining detailed socioeconomic status indicators confirm the robustness of our results. Single motherhood, low maternal education, and partner unemployment are linked to higher postpartum depression scores, while lack of health insurance and partner unemployment correlate with higher post-traumatic stress disorder symptoms. These findings highlight the multidimensional nature of socioeconomic disadvantage and its impact on maternal mental health.

Our findings have important implications for public health policies in Morocco and similar developing-country contexts. Routine screening for postpartum depression and post-traumatic stress disorder should be integrated into maternity care, particularly for women experiencing socioeconomic deprivation. Early identification and psychosocial support could mitigate the adverse consequences of maternal mental health disorders for both mothers and their children. Interventions targeting maternal mental health should incorporate socioeconomic support and address structural inequities. Evidence from the literature supports these recommendations. A scoping review of LMICs highlights the potential of integrating mental health services into existing primary healthcare, HIV, and maternal-child health programs to address the burden of common mental health disorders in resource-limited settings (40). A systematic review further emphasizes the importance of flexible, woman-centered perinatal mental health services delivered by trained professionals and supported by adequate organizational and structural resources (41).

### Strengths and limitations of the study

This study has several strengths, but also some limitations. It is among the few studies carried out in the context of developing countries, particularly in Africa, that examine the impact of socioeconomic vulnerability on maternal mental health. Although the sample size is relatively limited, the study is conducted across both public and private hospital centers, which reduces the risk of selection bias. Data are collected at the end of the maternity stay (immediate postpartum period), so our results specifically concern early postpartum mental health and may not capture the full trajectory of postpartum depression or post-traumatic stress symptoms that can develop later. In addition, some symptoms identified by the EPDS and PDI may overlap with transient emotional reactions (notably postpartum blues) and may therefore have been over(under)estimated. Self-reported measures such as the EPDS and PDI are subject to reporting bias, potentially leading to under(over)estimation of symptoms; however, these tools have been widely validated in the international literature. Socioeconomic deprivation is assessed using the EPICES score, a validated multidimensional instrument; we analyzed it both as a continuous variable and as a dichotomous indicator of vulnerability, and our findings are verified using classical socioeconomic indicators. While residual confounding by unmeasured variables cannot be completely eliminated, our analytical models considered all observable variables and accounted for most relevant factors reported in the literature to strengthen the validity of our findings.

## Conclusion

In this study, socioeconomic deprivation emerges as an important determinant of maternal mental health in the postpartum period, independently increasing the risk of both postpartum depression and post-traumatic stress disorder. These findings underscore the particular vulnerability of women in developing-country settings, where socioeconomic disadvantage compounds medical risks. Addressing maternal mental health therefore requires integrated strategies that go beyond clinical care to reduce socioeconomic inequalities and strengthen social protection for postpartum women. Further research is needed to better understand how socioeconomic disadvantage influences maternal mental health and to confirm these findings in other settings. Future studies should also evaluate interventions aimed at improving social support and maternal mental health outcomes.

## Data Availability

The datasets presented in this article are not readily available because access to the dataset is restricted. Data sharing is permitted only after obtaining prior administrative authorization from the participating hospitals and approval from the relevant Ethics Committee/Institutional Review Board. Therefore, the dataset cannot be made openly available and will be shared solely with authorized parties under these approvals. Requests to access the datasets should be directed to Saad Zbiri, szbiri@um6ss.ma.
