# Supplementary material for: Socioeconomic deprivation and maternal mental health in developing countries: a multicenter study of postpartum depression and post-traumatic stress disorder
Source: Front Glob Womens Health. 2026 Jul 30;7:1803491. doi: 10.3389/fgwh.2026.1803491 (PMC13469008; doi:10.3389/fgwh.2026.1803491)
Supplement: Supplementary file 1 [file Datasheet1.pdf]

Appendix

Table A1. Effect of socioeconomic deprivation on postpartum depression (full models)

Variables	Multivariate analysis: EPICES score	Multivariate analysis: Positive EPICES score
**Demographics**		
Age	0.034 (0.023)	0.027 (0.022)
Parity	0.040 (0.214)	-0.012 (0.191)
Children in the household	0.185 (0.239)	0.279 (0.219)
**Socioeconomic deprivation**		
EPICES score	0.038 (0.008)***	
Positive EPICES score		0.764 (0.406)*
**Medical history**		
Previous obstetric disorder	0.375 (0.269)	0.329 (0.261)
Previous mental disorder	1.845 (0.289)***	1.965 (0.284)***
Substance use	2.636 (1.193)**	2.967 (1.131)***
**Pregnancy**		
Multiple pregnancy	-0.776 (0.818)	-0.484 (0.773)
Abnormal fetal presentation	0.425 (0.451)	0.478 (0.429)
Pregnancy disease	0.274 (0.266)	0.088 (0.253)
**Delivery**		
Term at delivery		
Preterm	0.175 (0.473)	0.152 (0.467)
Full-term	reference	reference
Post-term	-0.524 (0.391)	-0.436 (0.372)
Mode of delivery		
Normal vaginal delivery	reference	reference
Instrumental vaginal delivery	-0.212 (0.603)	-0.114 (0.589)
Cesarean delivery	0.363 (0.539)	0.307 (0.524)
Episiotomy	0.839 (0.542)	0.678 (0.538)
Analgesia	-0.330 (0.419)	-0.386 (0.409)
Delivery complication	0.304 (0.363)	0.202 (0.347)
Private sector delivery	1.071 (0.483)**	0.307 (0.488)
**Early postpartum**		
Newborn weight		
Low	0.902 (0.531)*	0.900 (0.514)*
Normal	reference	reference
High	0.681 (0.591)	0.501 (0.555)
Newborn vitality		
Normal	reference	reference
Intermediate	-2.220 (1.207)*	-2.731 (1.275)**
Low	0.810 (0.780)	1.024 (0.778)
Artificial feeding	1.486 (0.454)***	1.304 (0.441)***
Maternal disease	1.227 (0.579)**	1.368 (0.551)**
Neonatal disease	0.076 (0.279)	0.183 (0.273)
N (observations)	527	527

Note: Coefficient estimates from logit models including all observables variables are presented with standard errors in parentheses.
Postpartum depression is assessed using the EPDS (Edinburgh Postnatal Depression Scale).

EPICES: socioeconomic deprivation index (*Évaluation de la Précarité et des Inégalités de santé dans les Centres d'Examens de Santé*).
*P-value < 0.10; **P-value < 0.05; ***P-value < 0.01

Table A2. Effect of socioeconomic deprivation on post-traumatic stress disorder (full models)

Variables	Multivariate analysis: EPICES score	Multivariate analysis: Positive EPICES score
**Demographics**		
Age	0.046 (0.024)*	0.040 (0.024)*
Parity	-0.279 (0.206)	-0.302 (0.211)
Children in the household	0.288 (0.241)	0.347 (0.244)
**Socioeconomic deprivation**		
EPICES score	0.029 (0.008)***	
Positive EPICES score		1.125 (0.511)**
**Medical history**		
Previous obstetric disorder	-0.151 (0.285)	-0.143 (0.281)
Previous mental disorder	1.830 (0.317)***	1.929 (0.316)***
Substance use	3.408 (1.379)**	3.612 (1.322)***
**Pregnancy**		
Multiple pregnancy	-0.665 (0.889)	-0.555 (0.907)
Abnormal fetal presentation	0.295 (0.475)	0.406 (0.481)
Pregnancy disease	0.821 (0.267)***	0.700 (0.260)***
**Delivery**		
Term at delivery		
Preterm	-0.340 (0.533)	-0.328 (0.523)
Full-term	reference	reference
Post-term	-0.432 (0.408)	-0.399 (0.397)
Mode of delivery		
Normal vaginal delivery	reference	reference
Instrumental vaginal delivery	-0.150 (0.609)	-0.047 (0.606)
Cesarean delivery	0.485 (0.555)	0.467 (0.539)
Episiotomy	0.500 (0.557)	0.368 (0.556)
Analgesia	-0.579 (0.434)	-0.632 (0.430)
Delivery complication	0.349 (0.403)	0.240 (0.393)
Private sector delivery	3.906 (0.523)***	3.712 (0.590)***
**Early postpartum**		
Newborn weight		
Low	0.619 (0.616)	0.635 (0.603)
Normal	reference	reference
High	1.079 (0.630)*	0.958 (0.600)
Newborn vitality		
Normal	reference	reference
Intermediate	-0.772 (1.166)	-1.016 (1.163)
Low	2.250 (1.197)*	2.499 (1.217)**
Artificial feeding	-0.249 (0.541)	-0.392 (0.532)
Maternal disease	1.952 (0.604)***	2.098 (0.606)***
Neonatal disease	0.223 (0.294)	0.262 (0.292)
N (observations)	527	527

Note: Coefficient estimates from logit models including all observables variables are presented with standard errors in parentheses.
Post-traumatic stress disorder is assessed using the PDI (Peritraumatic Distress Inventory).
EPICES: socioeconomic deprivation index (*Évaluation de la Précarité et des Inégalités de santé dans les Centres d'Examens de Santé*).
*P-value < 0.10; **P-value < 0.05; ***P-value < 0.01

Table A3. Effect of socioeconomic status on postpartum depression and post-traumatic stress disorder (full models)

Variables	Multivariate analysis: Postpartum depression	Multivariate analysis: Post-traumatic stress disorder
**Demographics**		
Age	0.043 (0.026)*	0.090 (0.028)***
Parity	0.110 (0.222)	-0.242 (0.208)
Children in the household	0.088 (0.249)	0.097 (0.251)
**Socioeconomic status**		
Single mother	2.949 (1.206)**	-0.014 (1.347)
Uninsured mother	0.461 (0.328)	0.723 (0.356)**
Maternal education		
High	reference	reference
Intermediate	0.649 (0.615)	-0.753 (0.595)
Low	1.198 (0.616)*	0.467 (0.639)
Non-working mother	0.127 (0.439)	0.504 (0.479)
Non-working partner	1.135 (0.519)**	1.403 (0.537)***
**Medical history**		
Previous obstetric disorder	0.243 (0.306)	-0.144 (0.321)
Previous mental disorder	2.287 (0.334)***	2.203 (0.363)***
Substance use	1.030 (2.299)	3.219 (2.227)
**Pregnancy**		
Multiple pregnancy	-0.166 (0.874)	-0.803 (0.947)
Abnormal fetal presentation	0.253 (0.518)	0.703 (0.526)
Pregnancy disease	0.229 (0.305)	1.073 (0.306)***
**Delivery**		
Term at delivery		
Preterm	0.099 (0.545)	-0.812 (0.582)
Full-term	reference	reference
Post-term	-0.966 (0.514)*	-0.508 (0.495)
Mode of delivery		
Normal vaginal delivery	reference	reference
Instrumental vaginal delivery	0.052 (0.656)	-0.790 (0.694)
Cesarean delivery	0.697 (0.620)	-0.209 (0.662)
Episiotomy	0.776 (0.594)	0.471 (0.618)
Analgesia	-0.623 (0.475)	-0.183 (0.514)
Delivery complication	0.004 (0.428)	0.160 (0.464)
Private sector delivery	0.206 (0.524)	3.822 (0.590)***
**Early postpartum**		
Newborn weight		
Low	1.274 (0.615)**	1.238 (0.716)*
Normal	reference	reference
High	0.840 (0.611)	1.281 (0.684)*
Newborn vitality		
Normal	reference	reference
Intermediate	-2.833 (1.453)*	-1.006 (1.362)
Low	1.489 (0.912)	2.514 (1.374)*
Artificial feeding	1.728 (0.527)***	-0.041 (0.607)
Maternal disease	1.309 (0.694)*	1.083 (0.720)
Neonatal disease	0.119 (0.321)	0.279 (0.332)

N (observations)	460	460

Note: Coefficient estimates from logit models including all observables variables are presented with standard errors in parentheses.
Postpartum depression is assessed using the EPDS (Edinburgh Postnatal Depression Scale).
Post-traumatic stress disorder is assessed using the PDI (Peritraumatic Distress Inventory).
EPICES: socioeconomic deprivation index (*Évaluation de la Précarité et des Inégalités de santé dans les Centres d'Examens de Santé*).
*P-value < 0.10; **P-value < 0.05; ***P-value < 0.01
